# T2 mapping for knee cartilage degeneration in young patients with mild symptoms

**DOI:** 10.1186/s12880-022-00799-1

**Published:** 2022-04-18

**Authors:** Huiyu Zhao, Hongqiu Li, Shuo Liang, Xinyue Wang, Feng Yang

**Affiliations:** 1 Department of Radiology, Central Hospital Affiliated to Shenyang Medical College, No. 5, South Seven West Road, Tiexi District, Shenyang, 110024 Liaoning China; 2 The 2Th Department of Orthopedic Surgery, Central Hospital Affiliated to Shenyang Medical College, No. 5, South Seven West Road, Tiexi District, Shenyang, 110024 Liaoning China

**Keywords:** Knee cartilage degeneration, T2 mapping, Young patients, Mild symptoms

## Abstract

**Background:**

We aimed to analyze the distribution of knee cartilage degeneration in young patients with mild symptoms using quantitative magnetic resonance imaging (MRI) T2 mapping.

**Materials and methods:**

This study included sixty six patients (case group) and twenty eight healthy volunteers (control group). The participants underwent 3.0 T conventional MRI plus a multi-echo sequence. The cartilage of each participant was divided into twenty eight subregions. We then calculated the T2 mean values and standard deviation or median and quartile range for each subregion according to whether the normal distribution was satisfied. Besides, we employed Kruskal–Wallis test to determine the statistical differences of each subregion in the control group while the Mann–Whitney U test was used to define the statistical difference between the case group and the control group and between the control group and subjects aged less than or equal to 35 years in the case group.

**Results:**

In the case group, age of 30 male patients was 31.5 ± 9.3 and age of 36 female patients was 35.7 ± 8.3. In the two groups, the superficial central lateral femoral region exhibited relatively high T2 values (control/case group: 49.6 ± 2.7/55.9 ± 8.8), and the deep medial patellar region exhibited relatively low T2 values (control/case group: 34.2 ± 1.3/33.5(32.2, 35.5)). Comparison of the T2 values between the case and the control group demonstrated a statistically significant increase in nine subregions (P_1_ < 0.05) and there were five subregions in the case group with age ≤ 35 years (P_2_ < 0.05). In particular, the *p*-values for four subregions of the patellofemoral joint were all less than 0.05 (P_1_ = 0.002, 0.015, 0.036, 0.005).

**Conclusion:**

T2 values of patients were significantly different with values of healthy groups, especially in the superficial cartilage of the patellofemoral joint. It made T2 mapping helpful to early identify patients with knee cartilage degeneration.

## Background

Articular cartilage has limited self-reparative capacity. As a result, cartilage damage due to traumatic or non-traumatic injuries increases susceptibility to early osteoarthritis (OA) [1, 2]. Previous studies have shown that people with intense physical exercises such as athletes experience high rates of cartilage damage [3, 4]. Thus, there is a need to adjust training schedules to minimize high mechanical stress to vulnerable areas [5]. Whereas exercise has a favorable impact on sporting career and improved quality of life, minor trauma in young adults without high risk factors could go unnoticed. This results gradual knee cartilage injuries in young adults which might evolve into osteoarthritis over time. There is an increased incidence of OA due to an aging population, obesity, infection or joint stress. Whereas studies have evaluated pathological mechanisms of OA, curative strategies are insufficient [6, 7]. Therefore, there is a need to observe and utilize preventive measures for OA, especially in young adults. Routine MRI has been shown to be an effective tool in the evaluation of articular cartilage. However, due to the limits in morphologic cartilage imaging sequences, there is low sensitivity in detecting early cartilage degeneration [8–10]. Quantitative T2 mapping exhibits broad usage of articular tissues, especially the cartilage [11, 12]. T2 values can be calculated through obtaining the signal intensities at different echo times from a multi-echo sequence. T2 mapping can detect early biochemical changes such as decreased proteoglycan content, increased free water content as well as more disordered collagen fibers [8]. On the contrary, in healthy individuals, water molecules are bound together by a well-organized collagen fiber matrix and exhibit low T2 relaxation times as compared to cases with cartilage degeneration [13, 14]. One study has confirmed that a multi-echo sequence with routine MR sequences get a great improvement in sensitivity in the identification of early cartilage degeneration [15]. In addition to cartilage, T2 mapping also shows a great feasibility at the sacroiliac joints [16].

Previous studies evaluated patients with obvious OA symptoms [15, 17]. However, before the development to OA, there are often undetected perturbations that occur in the cartilage. Here, we evaluated T2 relaxation time of knee cartilage and the regions with changes in young adults with mild symptoms who had not developed to OA. We analyzed the distribution of knee cartilage degeneration in the young adults with mild symptoms against healthy young adults.

## Materials and methods

### Study groups

This was a prospective study approved by the hospital ethics committee. All the participants gave written informed consent. We included a total of 66 patients and 28 healthy volunteers as a control group.

A total of 32 volunteers underwent routine MRI sequences coupled with a multi-echo sequence in the hospital between November 2020 and February 2021. We included volunteers aged between 18 and 35 years, had a body mass index (BMI) range from 18.5 to 23.9 kg/m^2^, with an occupation or hobby (e.g., firefighters, fitness coach, etc.) that could not lead to knee overuse and had no history of pain or other unusual symptoms in the knee joint. Besides, the volunteers had no history of knee injury, knee surgery, knee infection, medication (e.g., quinolones, glucocorticoids, etc.) or other diseases (e.g., immune system diseases, etc.) that could affect cartilage and production of high quality MR images. Three volunteers were excluded due to poor quality MR images and one volunteer was excluded due to abnormal signal on images. In the end, 28 volunteers were included.

On the other hand, the case group initially included 77 subjects between October 2020 and March 2021. These patients had mild symptoms of the knee joint, but the results of specific tests which included physical examination and X-ray were negative. Then they all underwent routine MRI sequences coupled with a multi-echo sequence. We included patients with mild pain or any mild discomfort, aged between 18 and 50 years and a BMI ranging from 18.5 to 23.9 kg/m^2^. At the same time, the patients with history of knee injury, knee surgery, knee infection, medication (e.g., quinolones, glucocorticoids, etc.) or other diseases (e.g., immune system diseases, etc.) that could affect cartilage and poor quality MR images were excluded. Five patients were excluded due to poor quality MR images and six patients were excluded due to overBMI and history of medication. Finally, this study enrolled 66 patients in the case group.

### MR image acquisition

All the participants were examined using 3.0 T MR (Siemens Magnetom Spectra; Siemens Healthcare, Erlangen, Germany) with an eighteen-channel knee array coil. They were not allowed undergo high intensity exercises related to knee joint 24 h to the examination. The subjects were in supine position with the coil center aligned with the lower edge of patella. The long axis of the knee joint was paralleled with the long axis of the scanning table to minimize the influence of magic angle effect. The MR examination sequences were; sagittal T1-weighted spin-echo sequence (T1-sag), sagittal fat-suppressed proton density weighted turbo spin-echo sequence (fspd-sag), coronal fat-suppressed proton density weighted turbo spin-echo sequence (fspd-cor), transverse fat-suppressed proton density weighted blade sequence (fspd-tra), and sagittal multi-echo sequence (T2 map) (Table 1).

**Table 1:** Parameters of the magnetic resonance imaging protocol

Sequence	T1-sag	fspd-sag	fspd-cor	fspd-tra	T2 map
Repetition time (ms)	400	3000	3000	3400	1000
Echo times (ms)	12	37	39	49	13.8, 27.6, 41.4, 55.2, 69.0
Field of view (mm)	160	160	160	160	160
Distance factor (%)	20	20	20	20	20
Number of slices	20	20	20	20	20
Slice thickness (mm)	3	3	3	4	3
Phase oversampling (%)	80	80	80	25	0
Flip angle (deg)	90	140	170	120	180
Phase resolution (%)	70	80	70	100	100
Matrix	269 × 384	256 × 320	224 × 320	320 × 320	384 × 384
Imaging time (min:s)	1:48	2:42	2:24	2:33	5:19

### MR image evaluation

**Fig. 1:**
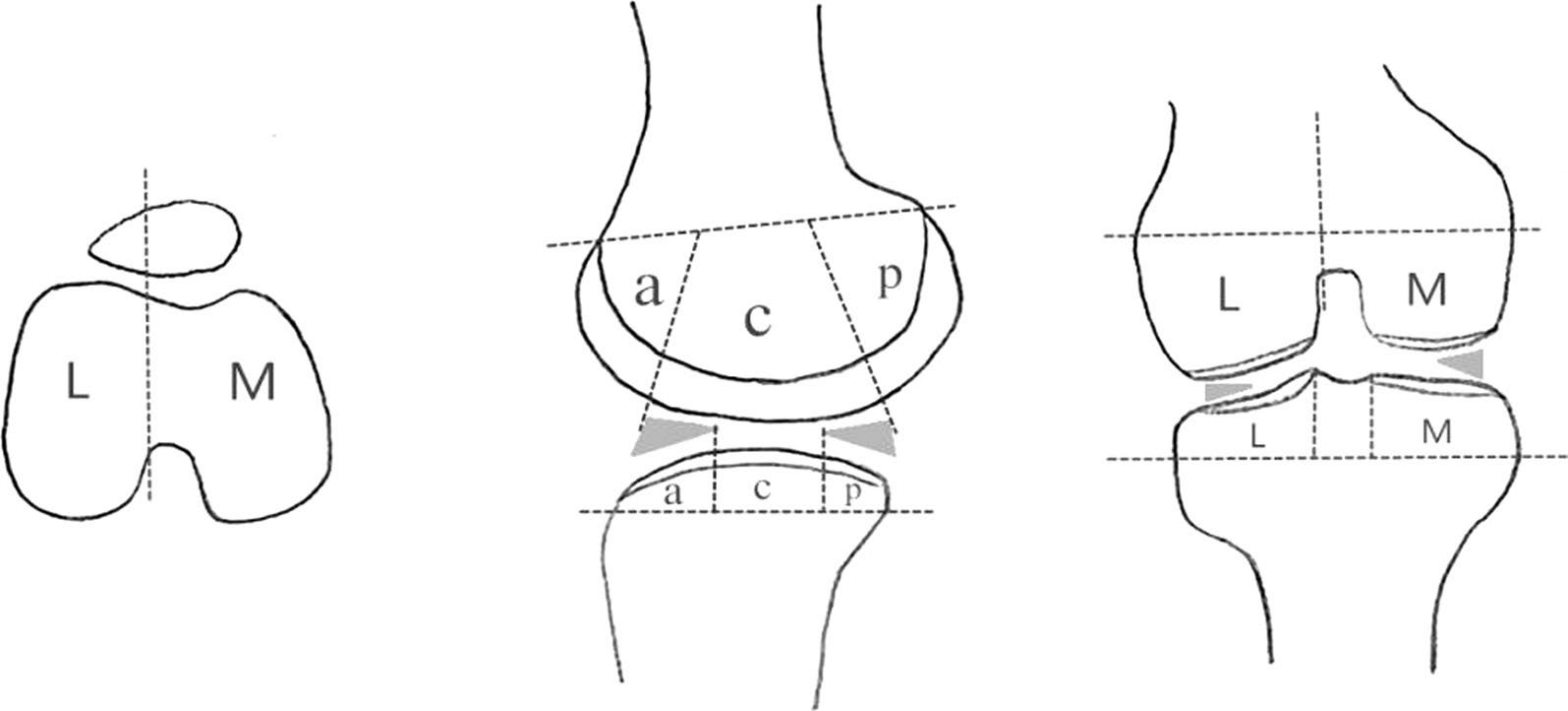
Regional subdivision of articular cartilage

**Fig. 2:**
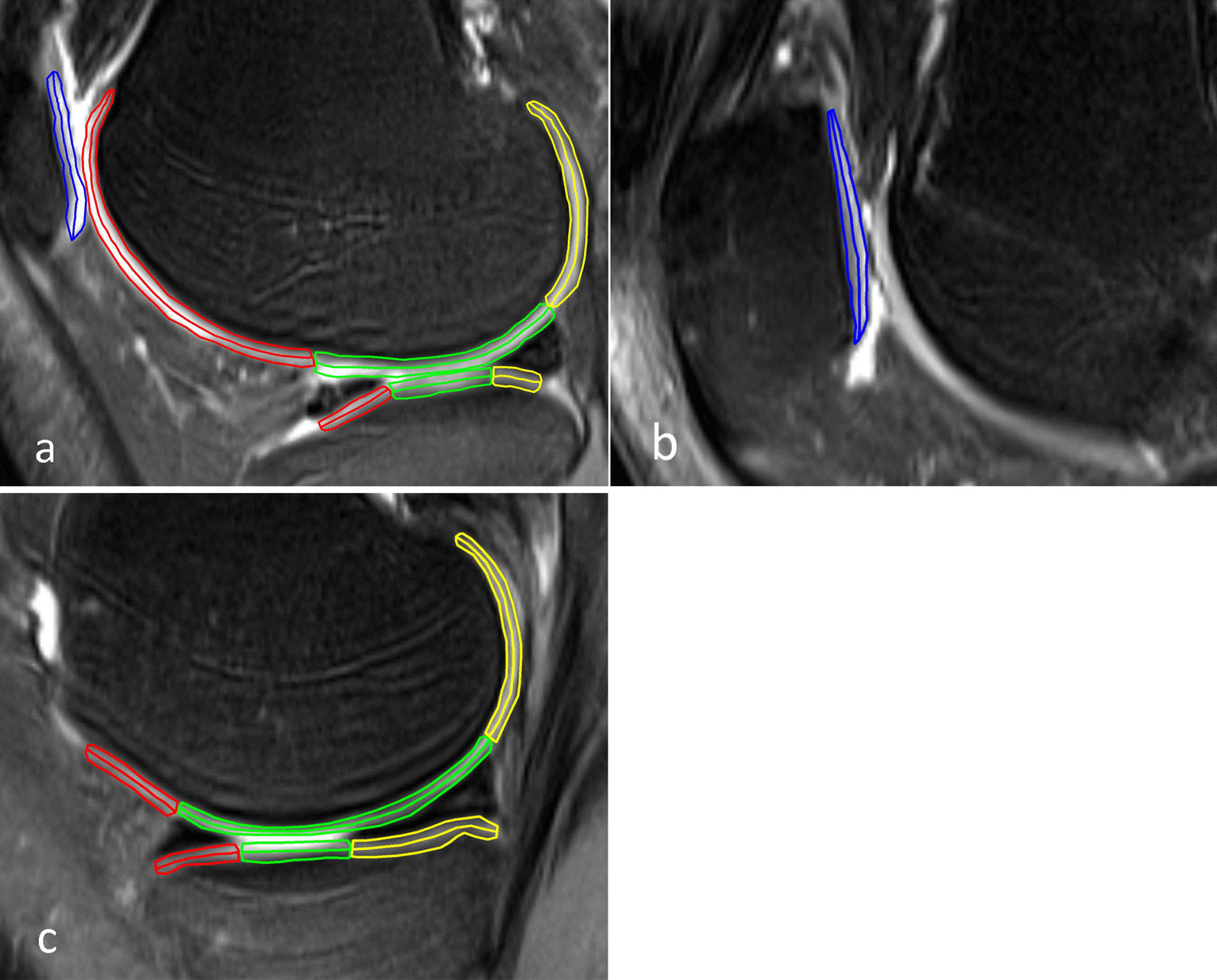
Sagittal fat-suppressed proton density weighted turbo spin-echo sequence images from the right knee cartilage of a 26-year-old male patient. Each region is equally divided into superficial and deep layer. Blue areas show lateral (**a**) and medial (**b**) patella cartilage. **a** and **c** illustrate lateral and medial cartilage of the tibiofemoral joint, respectively. Red, green and yellow areas represent the anterior, central and posterior cartilage, respectively. The ROIs size of this subject are 92, 105, 88, 102, 109, 102, 154, 148, 165, 168, 178, 183, 148, 133, 138, 130, 78, 83, 96, 98, 105, 94, 76, 77, 95, 97, 86, and 91 pixels (order according to Table 2)

All the images were processed using the Syngo workstation (Siemens Medical Systems). Images from the control group were assessed by a senior radiologist to ensure absence of abnormal signals, motion artifacts or other unusual changes. Thereafter, two resident physicians dissected T2 maps of the articular cartilage into measurement regions of interest (ROI) according to Whole-Organ Magnetic Resonance Imaging Score [18]. Images of two groups were stored in the same workstation in the order of examination time, so two physicians blinded to whether the images came from the case or control group. Figure 1 was the regional subdivision of articular cartilage. The patella (P) was divided into medial (M) with the ridge and lateral (L) regions. The ridge of the patella is the cartilage contact point location which occurred along the centerline. The femur (F) and tibia (T) were divided into medial (M) with the trochlear groove and lateral (L) regions. Both the femur and tibia were further divided into anterior (a), central (c) and posterior (p) regions. The central femur referred to the region extending from the anterior edge of the anterior horn to the posterior edge of the posterior horn of the meniscus. The medial tibia encompassed the region that was not covered by the anterior and posterior corners of the meniscus while the lateral tibia included the region that was covered by the body of the meniscus. To make the data more profound, each region was equally divided into superficial (s) and deep (d) layers according to the thickness of cartilage. The superficial area is from the articular surface to the middle of the cartilage and the deep area is from the middle of the cartilage to the bone-cartilage interface [5]. Together, the cartilage of the knee joint was divided into twenty-eight subregions (Fig. 2 and Table 2). Each ROI was measured two times to obtain average T2 values. The selected layer had clear cartilage boundary with each subregion displayed to the greatest extent. Similarly, the T2 maps of the case group were measured using the same protocol. However, the images or the outcome of the conventional sequences were not evaluated before measuring the ROI of T2 maps.

**Table 2:** mean values ± standard deviation or median (1st quartile, 3rd quartile) of T2 Values for the ROIs

Subregions			Control Group	Case Group
Medial Patella	Deep	MPd	34.2 ± 1.3	33.5 (32.2, 35.5)
	Superficial	MPs	35.5 (34.2, 36.5)	38.1 (35.2, 44.2)
Lateral Patella	Deep	LPd	35.8 ± 2.5	34.5 (33.1, 37.4)
	Superficial	LPs	37.7 ± 2.4	39.2 (36.9, 45.6)
Medial Femur	Anterior deep	MFad	41.0 ± 4.1	42.9 ± 6.6
	Anterior superficial	MFas	43.1 ± 4.2	45.8 ± 7.3
	Central deep	MFcd	43.0 ± 3.0	47.4 ± 8.9
	Central superficial	MFcs	44.2 (42.6, 47.5)	54.0 ± 12.0
	Posterior deep	MFpd	47.0 (44.5, 47.9)	48.6 ± 7.6
	Posterior Superficial	MFps	49.2 (48.0, 50.0)	52.1 (46.2, 59.0)
Lateral Femur	anterior deep	LFad	46.0 ± 2.8	47.8 (43.4, 54.3)
	Anterior superficial	LFas	48.3 ± 2.4	54.5 (46.6, 61.6)
	central deep	LFcd	47.7 ± 2.7	49.8 (46.0, 54.5)
	Central superficial	LFcs	49.6 ± 2.7	55.9 ± 8.8
	Posterior deep	LFpd	44.5 ± 4.3	46.5 (40.7, 54.5)
	Posterior superficial	LFps	47.2 ± 3.7	51.8 (42.8, 59.7)
Medial Tibia	anterior deep	MTad	36.9 ± 3.0	38.3 (34.2, 42.7)
	Anterior superficial	MTas	39.4 ± 3.8	41.2 (36.5, 46.7)
	central deep	MTcd	37.5 (35.9, 42.0)	40.5 (36.1, 46.5)
	Central superficial	MTcs	39.8 (37.9, 43.1)	43.1 (39.7, 50.7)
	Posterior deep	MTpd	39.0 ± 4.2	41.1 ± 6.6
	Posterior superficial	MTps	41.7 ± 3.9	44.2 (39.0, 47.8)
Lateral Tibia	anterior deep	LTad	40.1 ± 4.7	41.4 ± 7.2
	Anterior superficial	LTas	41.9 ± 4.4	43.6 (38.3, 50.2)
	Central deep	LTcd	40.3 ± 4.4	42.1 ± 7.0
	Central superficial	LTcs	42.2 ± 4.4	45.3 ± 8.5
	Posterior deep	LTpd	39.5 ± 4.5	41.1 ± 7.2
	Posterior superficial	LTps	42.1 ± 4.4	43.5 (37.4, 50.5)

### Statistical analysis

All the statistical analyses were performed using SPSS software (SPSS version 26.0, SPSS Inc.). Standard demographic data for both groups were reported as arithmetic mean values ± standard deviation. Inter-observer and intra-observer agreements were accessed using intra-class correlation coefficient (ICC). *P* values < 0.05 were considered statistically significant.

On the other hand, normal distribution of the data from the control group was first assessed by the Shapiro–Wilk test. If the data met normal distribution, it was represented as mean values ± standard deviation. Otherwise, the data was presented as a median (1st quartile, 3rd quartile). Due to skewed distribution, group difference among the ROIs was analyzed by Kruskal–Wallis test.

All the ROIs data in the case group were analyzed for normality of distribution using the Shapiro–Wilk test. Based on the results, the data were represented as mean values ± standard deviation or median (1st quartile, 3rd quartile). In addition, Mann–Whitney U test was used to evaluate the skewed distribution data from the same subregion between the two groups (P_1_ value) and between the control group and subjects aged less than or equal to 35 years in the case group (P_2_ value).

## Results

### Control group

We acquired MR images from the 28 volunteers with an average age of 27.0 ± 3.4. There was a total of 12 male volunteers (aged 23–33 years) with an average age of 26.8 ± 3.4 and 16 females (aged 24–35 years) with an average age of 27.1 ± 3.5. The average BMI was 21.1 ± 1.2 kg/m^2^ (range 18.7–23.4 kg/m^2^).

Our data showed that there were significant differences in T2 mean values among the subregions (*P* = 0.000). Deep layer T2 values were generally lower compared to the superficial layer (*P* = 0.000). Besides, the data showed significant differences in each region of the deep cartilage and superficial cartilage. The values of patella cartilage were lower than those of the femoral cartilage. In the patella cartilage, the T2 values of the medial regions were lower than those of the lateral regions. On the other hand, the T2 values of the medial region of the femur were higher in the central and posterior regions while in the lateral region, the T2 values were higher in the anterior and central regions. In the medial and lateral areas of the tibia, the T2 value was higher in the central and posterior regions (Tables 2 and 3).

**Table 3:** Comparison of internal regions in the control group

Groups	*P* value
All subregions	0.000
Patella subregions	0.000
Femur subregions	0.000
Tibia subregions	0.000
Anterior subregions	0.000
Central subregions	0.000
Posterior subregions	0.000
Patella deep subregions	0.016
Patella superficial subregions	0.001
Femur deep subregions	0.000
Femur superficial subregions	0.000
Tibia deep subregions	0.026
Tibia superficial subregions	0.047

### Case group

There was a total of 66 patients (aged 18–49 years) in the case group with an average age of 33.8 ± 9.0. There were 30 male patients (aged 18–48 years) with an average age of 31.5 ± 9.3 and 36 female patients (aged 25–49 years) with an average age of 35.7 ± 8.3. The average BMI was 22.0 ± 1.0 kg/m^2^ (range 18.7–23.9 kg/m^2^). Among them, 39 people are less than or equal to 35 years old.

**Fig. 3:**
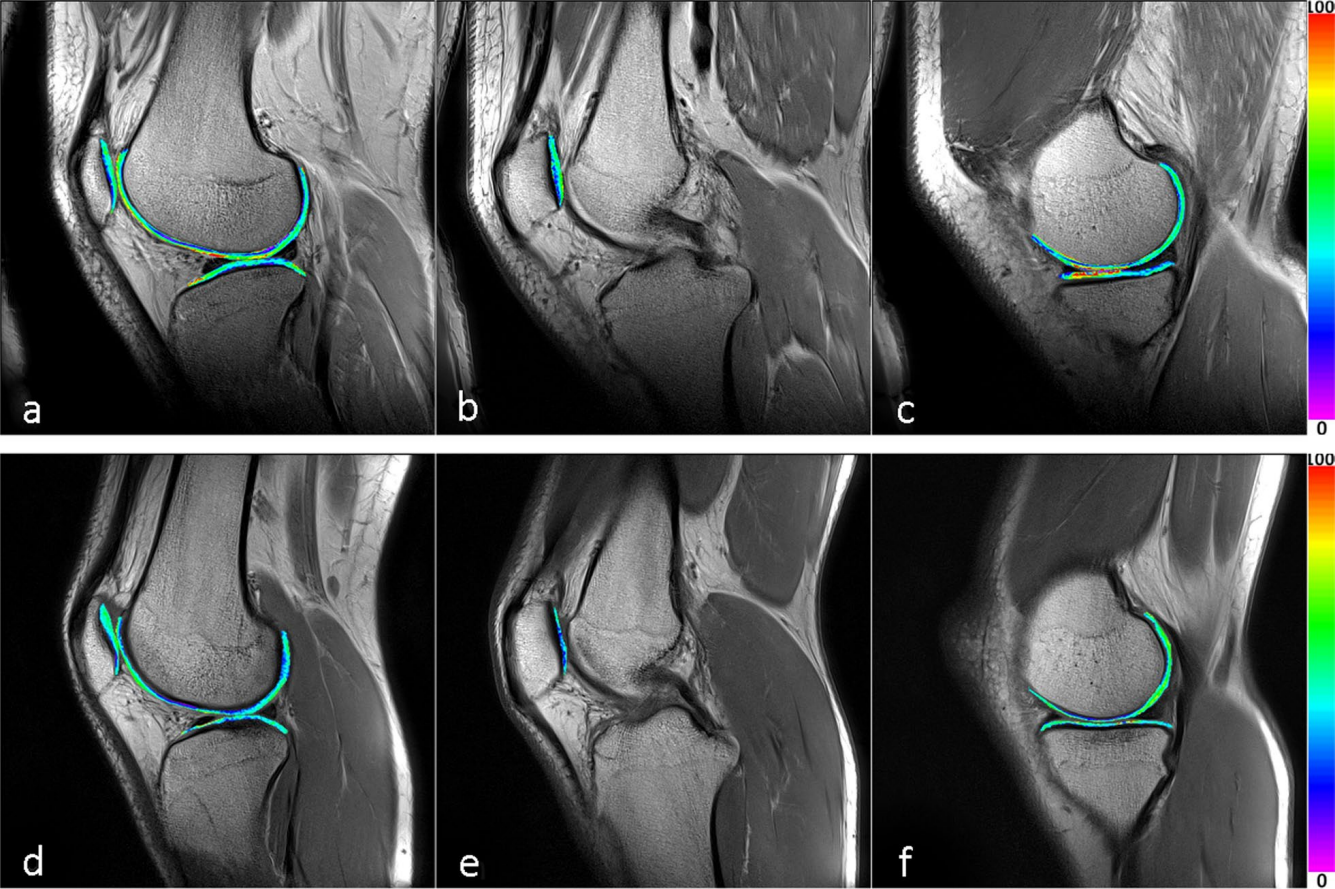
T2 maps of the right knee cartilage from a 26-year-old male patient (**a**–**c**) and the right knee cartilage from an 18-year-old male volunteer (**d**–**f**). **a** and **d** illustrate the lateral cartilage of tibiofemoral joint and patellofemoral joint. **b**, **c** and **e**, **f** illustrate the medial cartilage of the tibiofemoral joint and patellofemoral joint. The color scale is shown on the right

The T2 data demonstrated that nine subregions expressed statistically significant increase in the T2 values of the case group: MPs (P_1_ = 0.002), LPs (P_1_ = 0.015), MFas (P_1_ = 0.036), MFcd (P_1_ = 0.015), MFcs (P_1_ = 0.000), MFps (P_1_ = 0.021), LFas (P_1_ = 0.005), LFcs (P_1_ = 0.000) and MTcs (P_1_ = 0.011). There were five subregions in the case group with age ≤ 35 years: MPs (P_2_ = 0.009), LPs (P_2_ = 0.028), MFcs (P_2_ = 0.002), LFcs (P_2_ = 0.003) and MTcs (P_2_ = 0.018). In addition, the differences were more significant in the femur and patella regions. The data showed a significant difference in only one subregion of the tibia (Tables 2 and 4). We also showed the T2 maps of cartilage from one patient and one volunteer (Fig. 3).

**Table 4:** *P* value for the differences between the control group and case group

Subregion	P_1_ value	P_2_ value	subregion	P_1_ value	P_2_ value
MPd	0.229	0.736	LPd	0.175	0.127
MPs	0.002	0.009	LPs	0.015	0.028
MFad	0.183	0.269	LFad	0.085	0.088
MFas	0.036	0.111	LFas	0.005	0.062
MFcd	0.015	0.195	LFcd	0.094	0.162
MFcs	0.000	0.002	LFcs	0.000	0.003
MFpd	0.097	0.137	LFpd	0.298	0.751
MFps	0.021	0.083	LFps	0.069	0.186
MTad	0.179	0.611	LTad	0.357	0.689
MTas	0.149	0.225	LTas	0.256	0.844
MTcd	0.093	0.111	LTcd	0.401	0.353
MTcs	0.011	0.018	LTcs	0.132	0.104
MTpd	0.137	0.151	LTpd	0.307	0.839
MTps	0.112	0.084	LTps	0.112	0.100

### Intra-class correlation coefficient

There was significant inter-observer agreement between observer A and B (ICC = 0.946). Besides, the ICC values of observer A and B were highly reproducible (ICC_A_ = 0.980, ICC_B_ = 0.978).

## Discussion

We investigated the tibiofemoral joint and the patellofemoral joint cartilage degeneration based on the increase of T2 values in young adults with mild symptoms. Articular cartilage degeneration often develops into OA over time. However, before that, T2 mapping would give reliable hints for the patients.

T2 mapping has been used to evaluate articular cartilage T2 values in patients with early and late OA and then could distinguish the severity of OA based on magnetic resonance imaging grading. Previous studies have shown that T2 mapping could effectively monitor disease progression and assess treatment efficacy in OA [19]. This study included patients with mild symptoms. We aimed to probe measurable differences with healthy people in the cartilage. Recent studies have demonstrated that age and weight have a substantial impact on cartilage degeneration [20, 21]. The inclusion criteria were strictly controlled to minimize selection bias. The patients were advised to rest before examination to ensure that the outcome was meaningful and useful. Besides, high intensity exercise could lead to the deformation and disorientation of collagen fibers and promote the excretion of water molecules in the matrix, which could reduce T2 relaxation time [22]. In agreement with previous studies, our study demonstrated wider (31.2 ms to 57.5 ms) distribution of T2 values in the healthy control group [23, 24]. The T2 values were mainly associated with the scanning sequence of different equipment, the specific parameters of the sequence, as well as the magic angle effect [25–27]. This diversity resulted into the lack of unified standard T2 value range to judge the degeneracy of the cartilage. Some scholars considered that the reproducibility of T2 relaxation time quantitative measurements is a limitation. However, our study stated that T2 mapping of cartilage was highly reproducible. This also has been reported in other recent studies [28, 29].

**Fig. 4:**
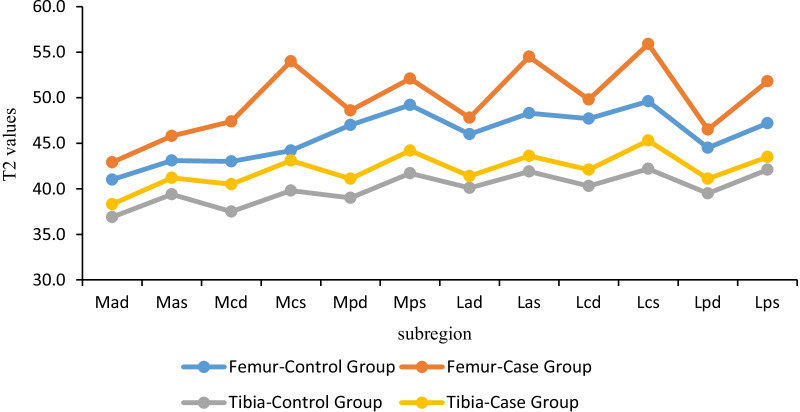
Line graphs are presented as mean values (or median, if not met normal distribution) of T2 Values for the ROIs

In this study, there were significant differences in T2 relaxation time in the different subregions. Based on the anatomy and locomotion mechanics, we divided the knee cartilage into fourteen regions. Considering the histological stratification of cartilage, each region was equally divided into two layers. So there were a total of twenty-eight subregions. The different regions of the knee cartilage were subjected to different mechanical strains during the daily activities. Our data showed larger T2 value of femur compared to that of the ipsilateral tibia, with an obvious load-bearing area of the middle femur. Besides, the lateral anterior region was larger than that in the medial femur. Figure 4 shows it clearly. The anterior femoral cartilage is a part of patellofemoral articular cartilage. Similar to the femoral cartilage of patellofemoral joint, the other part of the patella cartilage was higher on the lateral side than on the medial side. Contraction of quadriceps femoris produces two forces which pull the patella toward the femur to increase the pressure while the other causes shearing force in the proximal tibia relative to the distal femur [30]. Together, the data showed that the central load-bearing area bore the greatest stress. The lateral cartilage of the patellofemoral and tibiofemoral joints might also bear great stress. Previous studies have shown existence of a valgus angle in the physiological femoral and tibial joint lines in humans, and a considerable number of healthy people experience physiological varus of the knee joint once their bones are mature [31]. Varus and valgus have different load distribution on the cartilage [32]. Thus, future studies should explore the correlation between knee alignment and subregional T2 values of the femorotibial cartilage.

Similarly, our data showed that the patellofemoral cartilage was more prone to early cartilage degeneration with increasing age in the case group. In young patients, cartilage degeneration was mainly manifested in the superficial patella and central femoral regions. Besides, the superficial cartilage of the femur and patella was significantly different in the case group compared to that in the healthy participants. Figure 4 clearly displays it too. Whereas patients with mild pain of the knee often appear in the clinic, they don’t present obvious abnormality in physical examination, X-ray and even routine MRI analysis. Thus, T2 maps could dissect the possible diagnosis for the mild symptoms. On the other hand, through histological assays, changes in the collagen fiber ultrastructure and extracellular matrix, the cartilage can be divided into four layers; superficial, transitional, deep and the calcified zones [33]. However, Modl et al. demonstrated three articular cartilage layers on the T2 weighted images [34]. The cartilage from superficial to deep layers basically corresponds to the histological three-layer structure which are the superficial zone, the transitional zone and the deep calcified zones. Specific cartilage signal intensity of each depends on the pulse sequence [35]. The collagen layer structure can disappear due to cartilage degeneration in the early stage of development.

In people without obvious risk factors such as trauma, obesity, malnutrition or diabetes, combined with area vulnerability, it is feasible to speculate those improper behaviors might lead to early cartilage degeneration. The early stage presents a promising therapeutic window. Early behavior modification in the step-by-step treatment interventions is highly recommended. It has been shown that if patients change their bad living and working habits, they could strengthen the muscle strength training around the joints. This would not only improve joint stability, but promote local blood circulation [36–38]. The value of early diagnosis of joint cartilage degeneration is still debated. However, T2 mapping, as an aid tool to increase the diagnostic performance of MRI to identify patients with rheumatologic conditions, help to reduce the delay in diagnosis and to anticipate therapy [39, 40]. Elusive criteria is also a problem. There is a need for standardized imaging parameters, post-processing details and measurement methods to further improve the significance of the T2 value. Longitudinal studies by Verschueren et al. indicated that evaluation on the same scanner leads to reliable T2 mapping of changes in the cartilage [41].

Whereas our study present novel findings, there are several limitations to the study. Due to technical difficulties, we could only draw ROI manually. And cartilage could not be evaluated the differences of each layer in histology. In addition, our current study did not take the effect of knee alignment on cartilage T2 values into account. Because the cohort was relatively too small to conduct a comparative study on the differences in knee alignment between two groups. Most importantly, no follow-up of the patients was performed. There is a need for longitudinal follow-up to determine the clinical significance of the observed findings. Of course, the limitations also include our relatively strict inclusion criteria might have introduced minor selection bias. There remain some disadvantages, including no scoring of cartilage in the conventional MRI images and the lack of surgical and histological correlation.

Taken together, our data demonstrate that there is knee cartilage degeneration in patients with mild symptoms compared with healthy young adults and the distribution of knee cartilage degeneration is characteristic, especially superficial patellofemoral articular cartilage. In short, based on our findings, T2 mapping could be a suitable tool for routine articular cartilage examination, which would identify early cartilage degeneration.

## Data Availability

The datasets generated and analyzed during the current study are not publicly available due to future analysis plans but are available from the corresponding author on reasonable request.

## Abbreviations

OA: Osteoarthritis
BMI: Body mass index
MRI: Magnetic resonance imaging
ROI: Regions of interest
ICC: Intra-class correlation coefficient
